# Pain Perception Is Increased in Congenital but Not Late Onset Blindness

**DOI:** 10.1371/journal.pone.0107281

**Published:** 2014-09-22

**Authors:** Hocine Slimani, Sabrina Danti, Maurice Ptito, Ron Kupers

**Affiliations:** 1 Chaire de recherche Harland Sanders en Sciences de la vision, École d'Optométrie, Université de Montréal, Montréal, Canada; 2 BRAINlab, Department of Neuroscience and Pharmacology, Panum Institute, University of Copenhagen, Copenhagen, Denmark; Birkbeck, University of London, United Kingdom

## Abstract

There is now ample evidence that blind individuals outperform sighted individuals in various tasks involving the non-visual senses. In line with these results, we recently showed that visual deprivation from birth leads to an increased sensitivity to pain. As many studies have shown that congenitally and late blind individuals show differences in their degree of compensatory plasticity, we here address the question whether late blind individuals also show hypersensitivity to nociceptive stimulation. We therefore compared pain thresholds and responses to supra-threshold nociceptive stimuli in congenitally blind, late blind and normally sighted volunteers. Participants also filled in questionnaires measuring attention and anxiety towards pain in everyday life. Results show that late blind participants have pain thresholds and ratings of supra-threshold heat nociceptive stimuli similar to the normally sighted, whereas congenitally blind participants are hypersensitive to nociceptive thermal stimuli. Furthermore, results of the pain questionnaires did not allow to discriminate late blind from normal sighted participants, whereas congenitally blind individuals had a different pattern of responses. Taken together, these results suggest that enhanced sensitivity to pain following visual deprivation is likely due to neuroplastic changes related to the early loss of vision.

## Introduction

In a recent study we showed that congenitally blind individuals have reduced thresholds to heat and cold pain, and rate supra-threshold nociceptive stimuli as more painful compared to normally sighted individuals [1]. In sharp contrast, thresholds for innocuous cold and warmth perception were not altered, suggesting a specific effect for noxious thermal processing. These results add to a growing body of evidence that vision may affect pain processing [2]–[8]. The purpose of this study is to examine whether the loss of vision later in life also causes a hypersensitivity to pain.

There is abundant evidence from animal experiments that visual deprivation from birth causes dramatic plastic changes in the structural and functional organization of the visual cortex. The extent of these neuroplastic changes depends more strongly on the onset than on the duration of visual deprivation [9]–[12]. These findings have been corroborated by recent behavioral and brain imaging studies in humans showing that early blindness leads to compensatory plasticity and to a reorganization of the visual cortex [13], [15]. In sharp contrast, studies on late blindness have led to conflicting results. Whereas some studies showed that late blind individuals do not differ from normally sighted controls in various sensory and cognitive tasks [15]–[22], other studies indicated that late blindness also leads to sensory compensation and cross-modal plasticity [23]–[27].

To investigate whether late blind individuals also show hypersensitivity to nociceptive stimulation, we compared thermal pain thresholds and supra-threshold pain ratings of late blind (LB), congenitally blind (CB) and normally sighted (NS) volunteers. Participants also had to answer questionnaires regarding attention and anxiety towards painful encounters in daily life, since these factors are known to influence pain perception [28]. Based on our previous results in congenitally blind individuals [1] and the results by Zubek and colleagues [29] showing that prolonged visual deprivation leads to increased sensitivity to pain, we hypothesized that LB would also show increased pain sensitivity.

## Methods

### Participants

Participants were recruited from our database of congenitally and late blind subjects or by advertisement. Our total study population consisted of 23 CB (7F; mean age: 38.7±12.5 years; range: 20–61), 12 LB (7F; mean age: 50.1±11.4 years; range: 25–63) and 48 NS (20F; mean age: 38.9±13.6 years; range: 20–66) volunteers of whom 18 NS and 18 CB were included in a previous study and their data reused [1]. The study samples used for each of our measurements are listed in Table 1.

**Table 1:** Study samples used for each of the measurements.

Measurement	Group	Gender	Age (years ± SD)
Detection thresholds	LB	4 m/7 f	49.6±11.9
	CB	15 m/6 f	38.7±11.7
	NS	18 m/16 f	38.1±12.9
Supra-threshold ratings	LB	4 m/5 f	47.7±12.1
	CB	14 m/6 f	37.7±12.6
	NS	16 m/7 f	38.7±14.9
Pain questionnaires	LB	4 m/5 f	47.7±12.1
	CB	14 m/6 f	37.7±12.6
	NS	21 m/14 f	38.0±13.3

We calculated a blindness duration index (BDI) according to the formula “(age-age onset blindness)/age”. The BDI score can vary from 0 to 1, expressing the relative amount of time a person has been blind, with low scores indicating recent onset of blindness and high scores long duration of blindness. All blind participants suffered from blindness due to peripheral origin (retina, optic nerve). In the LB group, the average onset of blindness was 19.7±14.5 years and the average BDI was 0.6±0.3. Blindness due to diabetic neuropathy was an exclusion criterion. None of the participants suffered from known neurological or psychiatric disorders that might interfere with the experiment's results. Demographic details on the blind participants are provided in Table 2. All participants, including the blind, provided their written informed consent to participate in this study. The ethics committee for the city of Copenhagen and Frederiksberg, Denmark approved the study and the consent procedure.

**Table 2:** Demographics of the blind participants.

			Blindness		
ID	Age	Sex	Onset	Etiology	Residual vision
CB1	43	M	0	Retinoblastoma	-
CB2	39	M	0	Retinopathy of prematurity	Bright light
CB3	58	F	0	Retinopathy of prematurity	-
CB4	26	M	0	Retinopathy of prematurity	-
CB5	57	M	0	Retinopathy of prematurity	-
CB6	25	M	0	Retinopathy of prematurity	Bright light
CB7	37	M	0	Optic nerve atrophy	Bright light
CB8	21	M	0	Leber's amaurosis	-
CB9	25	M	0	Retinopathy of prematurity	-
CB10	58	M	0	Retinopathy of prematurity	-
CB11	42	F	0	Retinopathy of prematurity	-
CB12	34	M	0	Retinopathy of prematurity	-
CB13	49	M	0	Retinopathy of prematurity	-
CB14	36	F	0	Retinitis pigmentosa and bilateral macular perforation	Bright light
CB15	24	F	0	Retinopathy of prematurity	-
CB16	50	M	0	Retinopathy of prematurity	-
CB17	36	F	0	Retinopathy of prematurity	-
CB18	29	F	0	Retinopathy of prematurity	-
CB19	20	M	0	Unknown	-
CB20	61	F	0	Retinopathy of prematurity	-
CB21	36	M	3 mo	Unknown	-
CB22	43	M	1	Retinoblastoma	-
CB23	42	M	1	Meningitis	Bright light
LB1	55	M	6	Surgical accident	-
LB2	43	F	6	Retinopathy of prematurity	-
LB3	36	F	8	Glaucoma	-
LB4	44	M	9	Retinitis pigementosa	Bright light
LB5	56	M	10	Optic nerves sectioned by a bullet	-
LB6	56	F	10	Glass shards during accident	-
LB7	25	F	19	Taxoplasmosis	-
LB8	59	F	22	Iris infection	-
LB9	63	F	23	Retinitis pigementosa	-
LB10	48	F	32	Retinopathy of prematurity	Bright light
LB11	53	M	45	Meningitis	-
LB12	64	M	46	Retinitis pigmentosa	-

### Innocuous and noxious thermal thresholds assessment

We used a 3×3 cm Peltier-based thermotest (TSA-II, Medoc, Haifa, Israel) to determine thresholds for innocuous and noxious thermal stimuli on the dominant medial forearm. In order to reduce anxiety and fear, participants were familiarized with the thermal stimulation equipment and underwent practice trials prior to data acquisition. All participants were blindfolded after the familiarization period. The baseline temperature of the thermode was set to 32°C and we used a ramp rate of 1°C/s for the warmth and cool thresholds and 3°C/s for heat pain and cold pain thresholds. Stimuli were cued 2 to 5 s prior to onset. Participants had to click on a response key as soon as they detected warmth or cool or felt heat pain or cold pain. Thresholds were measured five times for each type of sensation with an inter-stimulus interval of 10–15 s for innocuous stimuli and 15–20 s for noxious stimuli.

### Supra-threshold pain ratings

We used a CO2 laser stimulator device with a spot diameter of 6 mm (LSD, SIFEC, Ferrières, Belgium) to generate highly accurate and contactless heat stimuli. This device is equipped with a contactless measurement unit with online monitoring of target skin temperature that controls the laser power in a closed-loop. This instant feedback guarantees that the skin is brought and maintained with a high accuracy at the exact target temperature, allowing the stimulation of the thinly myelinated Aδ- and the unmyelinated C-fibers without co-activation of the large myelinated Aβ-fibers [30]. Following an auditory cue, we applied stimuli of 3 s at 43, 45, 47 and 49°C on the dominant dorsal hand. Participants had to rate their sensation verbally on a 10-point rating scale with “0” as no pain, and “10” as the most intense pain imaginable. Each stimulus intensity was presented 3 times in a pseudo-randomized order with an interstimulus interval of 10 s. In order to avoid skin habituation or sensitization, the laser beam was moved after each stimulation following a 3×3 dots matrix. The dots were 1 cm apart from each other.

### Pain questionnaires

At the end of the session, participants filled in the Pain Vigilance and Awareness Questionnaire (PVAQ) adapted for a non-clinical population [31] and the Pain Anxiety Symptoms Scale (PASS) [32]. Both questionnaires comprise statements about pain encounters in everyday life. Participants had to rate at what frequency these situations apply to them. The PVAQ contains 16 items divided in 3 subscales: “Intrusion”, “Monitoring” and “Attention to changes in pain”. The PASS comprises 20 items divided into the 4 subscales “Physiological anxiety”, “Cognitive anxiety”, “Fear” and “Escape/Avoidance”. An audiotaped version of these questionnaires was presented to the blind participants.

### Statistical analysis

In order to estimate and account for the influence of demographic variables (i.e. gender, age), we conducted a multiple linear regression analysis that generated the regression models separately for threshold assessments and supra-threshold pain ratings. In each condition we modelled age and gender as independent and thresholds/supra-thresholds as the dependent variables. We obtained new supra-threshold/threshold values for each subject from the residuals of the multiple linear regression modeling. Data are presented as means ± SD.

We used Levene's test for assessing equality of variances of the data distributions for noxious and innocuous thermal threshold assessments (factor  =  “group” and dependent variable  =  “threshold”). Then, we conducted two-tailed Student t-tests in order to compare groups for noxious and innocuous thermal thresholds. For supra-threshold ratings, we conducted a 1-way ANOVA with the factor “group” as independent variable and “temperature” as dependent variable, checking for the equal of variances of the data distribution with a Levene test. Post-hoc comparisons were done using two-tailed Student t-tests, correcting for multiple comparisons (Bonferroni-Holm corrections, α = 0.05).

To investigate the effects of onset of blindness and blindness duration index on pain perception, we performed Pearson's correlations between these variables and pain thresholds and supra-threshold pain ratings in the LB group.

We performed the analysis of the PVAQ and PASS data using a principal component analysis (PCA; direct oblimin, δ = 0) on the questionnaires' raw scores to make a dimensionality reduction while preserving as much data variability as possible. Thereafter, we performed a Fisher Linear Discriminant Analysis (FLDA) on the resulting PCA factor scores to test whether LB, CB and NS responded differently. The variables were entered using the “all-variables together” method, while the goodness of classification analysis was tested using “leave-one-out” cross-validation and balanced for unequal sample sizes.

## Results

### Innocuous and noxious thermal detection thresholds

**Figure 1:**
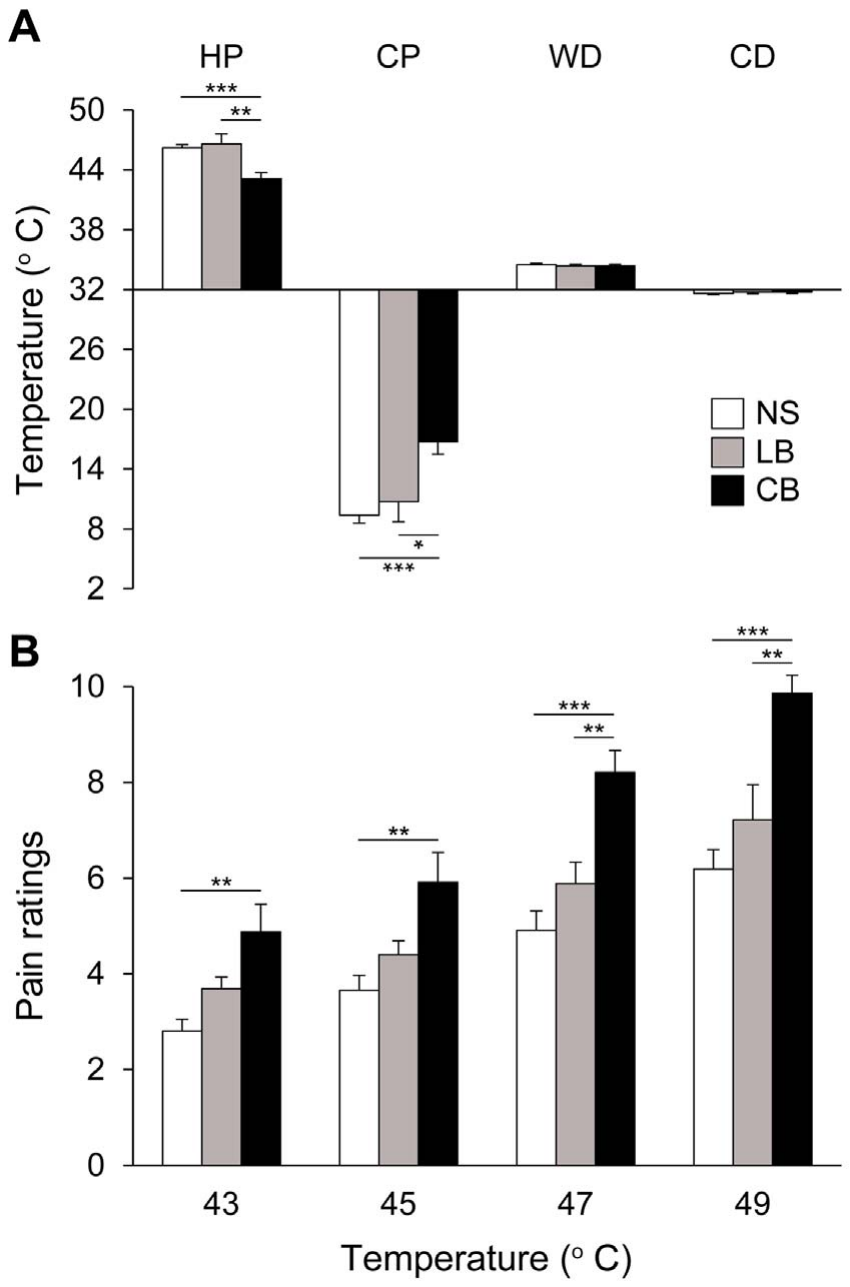
Thermal thresholds in normally sighted (NS), late blind (LB) and congenitally blind (CB) subjects. A: LB have heat pain (HP) and cold pain (CP) thresholds similar to NS. In contrast, HP and CP thresholds were significantly lower in CB compared to LB and NS. There were no group differences for innocuous warmth detection (WD) and cool detection (CD) thresholds. B: LB rate supra-threshold nociceptive stimuli similarly to NS. In contrast, CB rated supra-threshold stimuli as more painful compared to both LB and NS. Error bars represent the standard error of the mean. *p<0.05, **p<0.01, ***p<.001.

The Levene's tests indicated equality of variances of our data distributions (heat pain: F = 2.902, df_1_ = 2; df_2_ = 63, p = 0.062; cold pain: F = 2.587, df_1_ = 2; df_2_ = 63, p = 0.083; innocuous warmth: F = 2.165, df_1_ = 2; df_2_ = 63, p = 0.123; innocuous cool: F = 0.198, df_1_ = 2; df_2_ = 63, p = 0.821). Comparisons of pain thresholds (Figure 1A) failed to show differences between LB and NS for either heat pain (LB = 46.6±3.3°C, NS = 46.2±2.0°C; t = −0.49, df = 43, p = 0.628) or cold pain (LB = 10.8±6.8°C, NS = 9.4±4.7°C; t = −0.75, df = 43, p = 0.456). Importantly, compared to CB, LB had a significantly higher heat pain threshold (CB: 43.0±2.7°C; t = −3.3, df = 30, p = 0.003) and a lower sensitivity to cold pain (CB: 16.7±5.8°C; t = −2.5, df = 30, p = 0.015). As shown before, CB had a lower heat pain threshold (t = 5.0, df = 53, p<0.001) and were more sensitive to cold pain than NS (t = −5.1, df = 53, p<0.001). In contrast with the results of the pain thresholds, we found no group difference for innocuous warmth (NS = 34.5±0.8°C, CB = 34.4±0.7°C, LB = 34.4±0.5°C; LB vs NS: t = 0.5, df = 43, p = 0.642, LB vs CB: t = 0.03, df = 30, p = 0.981, CB vs NS: t = 0.6, df = 53, p = 0.559) and cold (NS = 31.6±0.6°C, CB = 31.7±0.6°C, LB = 31.7±0.6°C; LB vs NS: t = −0.7, df = 43, p = 0.515, LB vs CB: t = 0.09, df = 30, p = 0.933, CB vs NS: t = −0.9, df = 53, p = 0.361) detection thresholds. A Pearson correlation analysis indicated that the age of onset of blindness had no effect on heat pain (−0.238, p = 0.482) or cold pain (0.008, p = 0.981) thresholds. Likewise, BDI scores did not correlate with either heat pain (0.322, p = 0.334) or cold pain (−0.120, p = 0.726) thresholds.

### Supra-threshold pain ratings

The Levene's test indicated equality of variances of our data distributions (F = 2.193, df_1_ = 2; df_2_ = 49, p = 0.122). In line with the results of the pain thresholds, LB rated supra-threshold heat nociceptive stimuli (Figure 1B) similarly to NS (ANOVA: LB = 5.3±1.2, NS = 4.4±1.5; p = 0.110) and lower than CB (7.2±1.9; p = 0.011). As shown before, CB gave higher pain ratings than NS (p<0.001). More specifically, LB gave lower ratings than CB for the 47°C (LB = 5.9±1.4, CB = 8.2±2.1; t = 3.1, df = 27, p = 0.005) and 49°C (LB = 7.2±2.2, CB = 9.9±1.7; t = 3.6, df = 27, p<0.001) stimuli. Likewise, NSs pain ratings were lower than those of CB for the 43°C (NS = 2.8±1.2, CB = 4.9±2.6; t = −3.4, df = 41, p<0.001), 45°C (NS = 3.7±1.5, CB = 5.9±2.8; t = −3.3, df = 41, p = 0.002), 47°C (NS = 4.9±1.9; t = −5.4, df = 41, p<0.001) and 49°C (NS = 6.2±1.9; t = −6.6, df = 41, p<0.001) stimuli. Average pain ratings in LB did not correlate with either onset of blindness (0.149, p = 0.703) or BDI scores (−0.033, p = 0.933).

### Pain questionnaires

**Figure 2:**
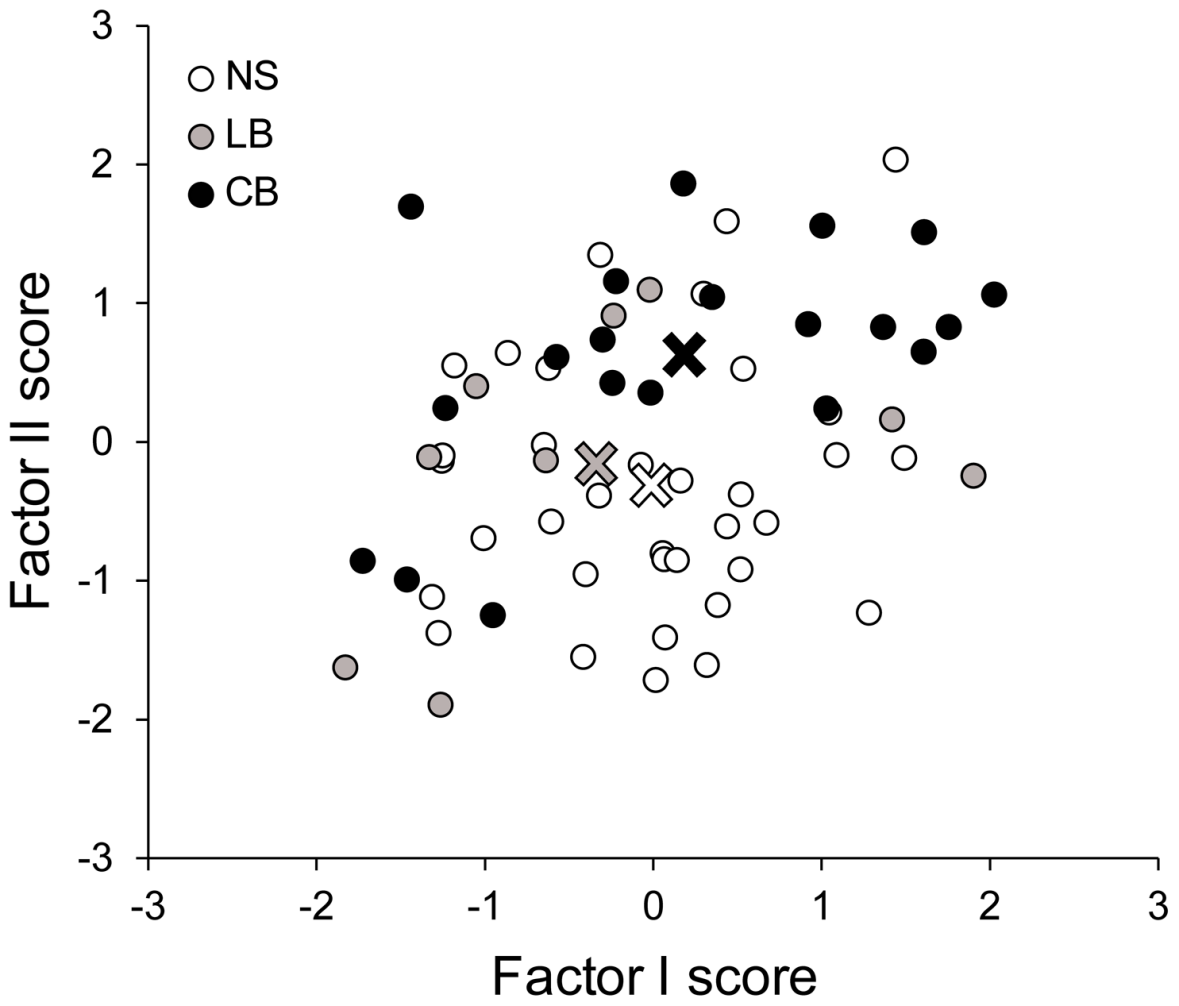
Principal component analysis of the Pain Anxiety Symptoms Scale (PASS) and Pain Vigilance and Awareness Questionnaire (PVAQ). Factors I (anxiety) and II (attention to pain) result from an oblique rotation. Higher values indicate higher correlation scores, with the average centered at 0. Color-coded crosses represent centroids after principal component analysis (PCA). LB have a similar responses pattern, whereas CB and NS show a distinct pattern of factor loadings, where factor II (attention to pain) discriminates better than factor I (anxiety). Normally sighted (NS), late blind (LB) and congenitally blind (CB) participants are represented with white, grey and black dots, respectively.

The Kaiser–Meyer–Olkin (KMO) measure verified the sampling adequacy of the PCA analysis we conducted (overall KMO = 0.75; KMO for each sub-factor >0.5). Bartlett's test of sphericity (χ^2^ = 237.6, Dof = 21, p<0.001) indicated that correlations between PVAQ and PASS sub-factors were sufficiently high for PCA. Two components had eigenvalues over Kaiser's criterion of 1 and in combination explained 72.5% of the variance. Thereafter, we performed a FLDA to classify the participants on the basis of their regression factor scores derived from the PCA analysis. As illustrated in Figure 2, this analysis indicated that LB and NS had an undistinguishable response pattern, as we obtained a classification accuracy of only 54.1 % (chance level  = 50 %; canonical r^2^ = 0.969, χ^2^ = 1.3, Dof = 2, p = 0.521). On the other hand, the FLDA allowed us to correctly discriminate CB from NS with an accuracy of 75.3 % (canonical r^2^ = 0.788, χ^2^ = 12.4, Dof = 2, p = 0.002). Since the above classification scores were mainly driven by factor II (attention to pain), we infer that CB are more attentive to signals of threat than NS. Inversely, the poor contribution of factor I (anxiety) suggests that CB are not more anxious than NS about pain encounters in daily life. Pearson correlation analysis within LB indicated that the onset of blindness had no effect on either factor I (−0.294, p = 0.443) or factor II (−0.504, p = 0.167). Likewise, BDI scores also did not correlate with either factor I (−0.026, p = 0.948) or factor II (0.423, p = 0.257).

## Discussion

The purpose of the present study was to investigate if individuals with acquired blindness show thermal hypersensitivity to noxious thermal stimulation as previously reported in congenital blindness [1]. In contrast with our hypothesis, late blind and sighted participants showed similar heat and cold pain thresholds and supra-threshold pain ratings. This indicates that onset of blindness, and not blindness per se, is the driving factor of thermal pain hypersensitivity in individuals lacking vision.

Our findings are in line with previous studies indicating that late blind individuals show no compensatory plasticity for auditory [15], [16] or tactile [15], [21], [22] information processing. Indeed, the extent of cortical reorganization strongly depends on the onset of visual deprivation, as many animal and human studies have shown that structural and functional brain changes following blindness are less likely to occur later in life [14], [33]. However, these data need to be interpreted with some caution due to our medium-sized study sample of late blind individuals, our results further show that there was no correlation between pain perception and the blindness duration index or onset of blindness, indicating that individuals who have lost their vision relatively early do not differ in pain responsiveness from those who have lost their vision later in life. It should be noted that the earliest onset of blindness in our LB group was six years of age, which is possibly after the critical period during which absence of vision affects nociceptive processing. In support of this, studies have shown that the switch of body coordinates from anatomical to external frame of reference takes place before the age of six [34]. It has also been demonstrated that touch perception is hampered by conflicting inputs from anatomical and external body frames of reference in sighted [35]–[40] and late blind [37], but not congenitally blind individuals [37], [41]. Furthermore, there is evidence that pain perception is also affected by body frame of reference and body representation [9], [42], [43], [44],

Our psychophysical data are further corroborated by the psychometric results that also failed to find differences in attitude and responses to signals of threat in daily life between LB and NS. Indeed, results of the pain questionnaires indicated that LB and NS pay similar attention to environmental threats and react with the same level of anxiety to such threats. In sharp contrast, CB scored higher than NS on attention to pain. This increased awareness of potentially dangerous stimuli could partly explain the increased pain responsiveness in CB since attention is known to exacerbate the experience of pain [45]. This suggests that CB allocate more attentional resources to potentially threatening stimuli in order to avoid or reduce pain. This finding is in accordance with a recent study showing that CB are hyper-responsive to threatening auditory stimuli, and that this was associated with stronger amygdalar activations [46]. This increased awareness of danger could compensate for the lack of vision that is necessary to quickly adopt optimal defensive and protective behaviors [47].

Previous studies have shown that increased attention to threatening stimuli can be driven by augmented levels of anxiety [28]. One could therefore argue that the lack of vision may increase anxiety and consequently also attention towards nociceptive stimulation [1], [48]. However, our psychometric data rule out this possibility, as all three groups were equally anxious about environmental threats. Furthermore, since LB and NS did not differ in attention to pain, it seems that early blindness is necessary to develop increased attention to environmental threats.

In conclusion, we show that blindness acquired at the age of six or later does not lead to pain hypersensitivity. Our data therefore suggest that hypersensitivity to noxious stimulation is the result of neuroplastic changes that occur early in development. Whether attention is the chief determinant of the exacerbated sensitivity to pain in congenitally blind individuals, or simply a potentiating factor, needs further investigation.
